# Mitigating Sulfidogenesis With Simultaneous Perchlorate and Nitrate Treatments

**DOI:** 10.3389/fmicb.2018.02305

**Published:** 2018-10-04

**Authors:** Anna Engelbrektson, Vanessa Briseno, Yi Liu, Israel Figueroa, Megan Yee, Gong Li Shao, Hans Carlson, John D. Coates

**Affiliations:** Energy Biosciences Institute, University of California, Berkeley, Berkeley, CA, United States

**Keywords:** souring, sulfidogenesis, oil production, perchlorate, perchlorate reducing bacteria

## Abstract

Sulfide biogenesis (souring) in oil reservoirs is an extensive and costly problem. Nitrate is currently used as a souring inhibitor but often requires high concentrations and yields inconsistent results. Recently, perchlorate has displayed promise as a more potent inhibitor in lab scale studies. However, combining the two treatments to determine synergy and effectiveness in a dynamic system has never been tested. Nitrate inhibits perchlorate consumption by perchlorate reducing bacteria, suggesting that the combined treatment may allow deeper penetration of the perchlorate into the reservoir matrix. Furthermore, the metabolic intermediates of perchlorate and nitrate reduction (nitrite and chlorite, respectively) are synergistic with the primary electron acceptors for inhibition of sulfate reduction. To assess the possible synergies between nitrate and perchlorate treatments, triplicate glass columns packed with pre-soured marine sediment were flushed with media containing sulfate and an inhibitor treatment [(i) perchlorate; (ii) nitrate; (iii) perchlorate and nitrate; or (iv) none]. Internal geochemistry and microbial community changes were monitored along the length of the columns during six phases of increasing treatment concentrations. In a final phase all treatments were removed. Sulfide production decreased in all treated columns in conjunction with increased inhibitor concentrations relative to the untreated control. Interestingly, the potency of the “mixed” treatment was additive relative to the individual treatments suggesting no interaction. Microbial community analyses indicated community shifts and clustering by treatment. The mixed treatment column community’s trajectory closely resembled that of the community found in the perchlorate only treatment, suggesting that perchlorate was the dominant control on the “mixed” community structure. In contrast, the nitrate and untreated column communities had unique trajectories. This study indicates that concurrent nitrate and perchlorate treatment is not more effective than perchlorate treatment alone but is more effective than nitrate treatment. As such, treatment decisions may be based on economic factors.

## Introduction

Hydrogen sulfide production in oil systems is a costly and potentially dangerous problem leading to pipeline and equipment corrosion and potential failure. A number of different treatments are currently used to inhibit *in situ* sulfide production, known as souring, in oil reservoir systems. These treatments include the use of low sulfate injection water or water from which sulfate has been removed, chemical biocides to limit overall microbial growth, and nitrate treatment (Gieg et al., 2011). Of these methods nitrate is the only method that specifically targets sulfate reducing microorganisms (SRM).

Nitrate has been used as a biological treatment in oil reservoir systems since the 1990s (Coates et al., 1993; Hubert et al., 2003; Arensdorf et al., 2009; Gieg et al., 2011). This treatment has direct and indirect effects on sulfate reduction but is not always predictable (Hubert and Voordouw, 2007; Voordouw et al., 2009; Gieg et al., 2011; Carlson et al., 2015a; An et al., 2017; Okpala et al., 2017; Suri et al., 2017; Engelbrektson et al., 2018). Nitrate has multiple mechanisms of action to control sulfate reduction. As one of the most thermodynamically favorable electron acceptors, nitrate reduction is a far more favorable than sulfate reduction resulting in biocompetitive exclusion of the sulfate reducing organisms, presuming they are competing for the same electron donor. Additionally incomplete nitrate reduction can form the intermediate nitrite, which is highly toxic to sulfate reducing bacteria. Nitrate reduction linked to sulfide oxidation can also produce elemental sulfur or sulfate depending upon the strain (Gevertz et al., 2000). Perchlorate treatment represents an emerging technology as a specific inhibitor of biological sulfate reduction and has been demonstrated to be effective in both batch and continuous flow systems (Engelbrektson et al., 2014, 2018; Gregoire et al., 2014; Carlson et al., 2015a). Additionally, Perchlorate is effective at lower concentrations compared to nitrate, and appears more predictable and more consistent in its effect than nitrate (Engelbrektson et al., 2014, 2018; Carlson et al., 2015a). Perchlorate is both a direct and indirect inhibitor of sulfate reduction. It is a direct inhibitor of the enzymes required for sulfate reduction and is an indirect inhibitor in that, like nitrate reduction, perchlorate reduction is energetically more favorable than sulfate reduction with an E^o′^ = + 797 mV versus E^o′^ = -217 mV for sulfate (Youngblut et al., 2016). Additionally all dissimilatory perchlorate reducing organisms have the ability to oxidize sulfide to elemental sulfur with no associated energy gain (Gregoire et al., 2014; Mehta-Kolte et al., 2017).

A small number of past studies have investigated the use of mixtures of two compounds to combat souring. Greene et al. (2006) investigated mixtures of various biocides and nitrite on a microbial consortium and Carlson et al. (2015a) studied the effectiveness of mixed nitrate and perchlorate treatment, but only on the pure culture *Desulfovibrio alaskensis* G20 in batch systems. This pure culture study indicated that the inhibition is additive in batch culture but is tending toward antagonism (Carlson et al., 2015a) demonstrating that the two compounds have similar mechanisms of action. Subsequent biochemical and molecular studies confirmed that the common inhibitor target was the ATP sulfurylase enzyme, a prerequisite of sulfate reduction and is conserved across all SRM (Carlson et al., 2015a). Additionally, perchlorate is synergistic with nitrite while nitrate is synergistic with chlorite suggesting that metabolic intermediates of the individual respiratory metabolisms could mediate a synergistic effect of combined treatments. However, a mixture of these two particular treatments in a dynamic community system has never been investigated and is of particular interest as many oil fields are currently undergoing nitrate treatment.

In the majority of perchlorate-reducing organisms, the presence of nitrate in the growth media inhibits perchlorate reduction by impacting the expression regulation of the metabolic pathway (Chaudhuri et al., 2002; Coates and Achenbach, 2004; Sun et al., 2009; Wang et al., 2018). However, in a few exceptional cases, such as *Sedimenticola selanatireducens* CUZ, nitrate and perchlorate are used simultaneously when the culture is pre-grown on nitrate, while perchlorate is preferentially used when the culture is pre-grown on perchlorate (Carlstrom et al., 2015). In communities, perchlorate reduction often does not occur until after the nitrate has been completely consumed (Coates and Achenbach, 2004; Nozawa-Inoue et al., 2005, 2011; Choi and Silverstein, 2008; Coates and Jackson, 2009). However, this isn’t a universal phenomenon. Zhao et al. (2011) demonstrated that the two electron acceptors could be simultaneously reduced in a hydrogen based membrane biofilm reactor, but the removal percentage depends upon the amount of nitrate and electron donor present implying that nitrate remains the preferred substrate. On the assumption that nitrate is preferentially respired before perchlorate in an oil reservoir system, then combining the treatments could push perchlorate further into the reservoir matrix allowing the mixture to be more effective than the individual inhibitors alone and extend the zone of their activity. The stratification of nitrate and perchlorate reduction could create a dual barrier to the rebound of sulfidogenesis. The microbial communities involved in nitrate and perchlorate reduction are distinct (Engelbrektson et al., 2014, 2018) and the mechanism of reactive chlorine species and reactive nitrogen species toxicity to SRM are different. As such, emergence of a resistant SRM population would require co-evolution of resistance to both nitrate and perchlorate reducing microbial communities and their associated changes in the environmental geochemistry.

This investigation examined the effectiveness of mixed treatment using perchlorate and nitrate compared to the individual treatments alone in a dynamic flow packed column system. We investigated the hypothesis that the addition of nitrate to perchlorate would increase the zone of inhibitor impact and that the two treatments combined would yield an additive effect. This was done by increasing the total inhibitor concentration in each column over time and monitoring the geochemistry across the columns. Additionally, we explored the effect of mixed treatment on the microbial community compared to the effects of each treatment individually to identify the possible dominance of one inhibitor over the other.

## Materials and Methods

### Column Setup

**FIGURE 1:**
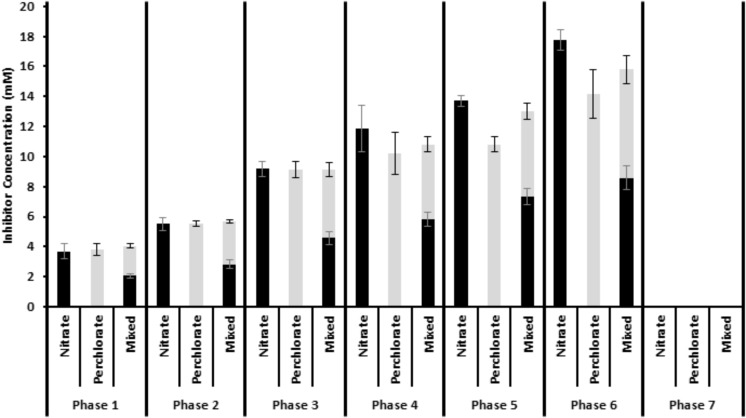
Influent inhibitor concentrations throughout the treatment phases. Nitrate is shown in black and perchlorate is shown in gray. The mixed treatment shows the combination of the two treatments. Error is standard deviation of weekly influent bottle measurements over the entire treatment phase.

Twelve one liter columns were packed with a pre-soured mixture of San Francisco bay water, San Francisco bay sediment, crude oil, and sand and secured on their sides (**Supplementary Figure S1**). The sediment was pre-soured by mixing all packing material in a bucket, adding yeast extract and incubating at room temperature for approximately 1 month. Sets of triplicate columns were fed through a peristaltic pump with autoclaved degassed medium. The medium (APM) consisted of 20 g/L NaCl, 0.67 g/L KCl, 2.5 g/L NaHCO_3_, 3.55 g/L Na_2_SO_4_, 10 ml vitamins, 10 ml minerals, 20 ml RST minerals (Balch et al., 1979; Carlström et al., 2013) along with a mix of volatile fatty acids to a final concentration of 1093.2 μM sodium acetate, 14.8 μM Formic Acid, 3.82 μM Butyric Acid, and 9.12 μM Propionic acid. After autoclaving, 30 mL each of MgCl_2_ x 6H_2_O (424 g/L) and CaCl_2_ × 2H_2_O (60.8 g/L) were added to the medium and it was degassed and kept under an 80/20 N_2_/CO_2_ headspace. The columns were allowed to stabilize until each column had equivalent sulfide generation. At this point (day 0) an inhibitor chemical [calcium nitrate, sodium perchlorate, or a 50:50 (mole:mole) mix of sodium perchlorate and calcium nitrate] was added to the media at varying concentrations throughout the seven phases of the study. The treatment phases ranged in length from 31 days to 63 days (**Figure 1**) and the treatment concentrations stepped up from an average concentration of 3.85 mM in phase 1 (nitrate: 3.67 ± 0.50 mM, 18.35 ± 2.50 electron equivalents; perchlorate: 3.83 ± 0.40 mM, 30.64 ± 3.2 electron equivalents; Both: 4.05 ± 0.21 mM, 26.26 ± 0.67 electron equivalents) to 15.91 mM in phase 6 (nitrate: 17.75 ± 0.37 mM, 88.75 ± 1.85 electron equivalents, perchlorate: 14.18 ± 1.61 mM, 113.44 ± 12.88 electron equivalents; Both: 15.8 ± 1.24 mM, 100.66 ± 8.47 electron equivalents) (**Figure 1**). In the seventh phase, treatment was suspended to assess re-souring. One triplicate set of columns was left untreated throughout all treatment phases. Influent sulfate concentrations varied very little throughout the experiment (23.41 mM ± 1.66 mM) and no sulfate was consumed in the lines between the media bottle and the column (**Supplementary Figure S2**).

Eluent flow through the columns was 1.52 ± 0.06 mL/h resulting in a calculated retention time of 9.04 ± 0.36 days. Samples were collected from 6 ports (ports 2–7) along the length of the columns weekly by pulling 2 mL of the column bed with a modified syringe (the end was clipped off to allow column bed material to enter the syringe). The collected samples were centrifuged in 2 mL capped centrifuge tubes for 1 min at 12,000 ×*g*. The resultant supernatant was filtered through a 0.2 μm nylon syringe filter for use in geochemical measurements and the pelleted material was immediately frozen on dry ice and stored at -80°C for later microbial community analysis. Influent samples were collected by removing approximately 10 mL from the medium reservoir and filtering (0.2 μM nylon filter) into a vacutainer tube (BD, Franklin Lakes, NJ, United States) for storage at 4°C. Port 1 samples are equivalent to influent samples at the point where they enter the column and 7 ml volumes were collected directly from the feed line and filtered (0.2 μM nylon filter) into a vacutainer tube for storage at 4°C.

### Geochemical Measurements

Sulfide concentrations were measured immediately after sampling using a modified Cline assay (Cline, 1969; Engelbrektson et al., 2014). Briefly, each sample was diluted with deionized water to bring them into a measurable range and read at 660 nm on a Varian Cary 50 Bio spectrophotometer equipped with a Cary 50 MPR microplate reader. Following this, sulfide was removed using iron and sodium hydroxide (Engelbrektson et al., 2014). Average cumulative sulfide in millimoles was calculated using the empirically determined eluent flow rate noted above. Average rates of sulfide production in millimoles per day were calculated for each treatment phase using the slopes from the cumulative data. These rates were then normalized to the average rate of sulfide production for the same phase from the control columns and expressed as “percent of control.”

Sulfate, nitrate, and perchlorate concentrations were measured using ion chromatography on a Dionex ICS-1500 with a Thermo Scientific Dionex IonPac AS25 Hydroxide-Selective Anion-Exchange Column and a 36 mM sodium hydroxide flow rate of 1 mL/min.

Volatile fatty acids (acetate, propionate, and butyrate) were measured with a modified liquid-liquid extraction (Banel and Zygmunt, 2011). Briefly, the pH of samples and VFA mix standards prepared in seawater were adjusted to <2 using concentrated sulfuric acid. VFAs were then extracted by adding 1.5 g of sodium sulfate and 1 mL of Methyl tert-butyl ether (MTBE) spiked with 50 μM acetic acid d_4_ (Sigma-Aldrich, United States) as internal standard. The mixture was vortexed for 5 min and allowed to settle. Approximated 0.7 mL of the top MTBE layer was transferred into GC-vial for analysis on gas chromatograph-mass spectrometry (GC-MS) using selective ion mode (SIM) with an Agilent BD-FFAP Column (length 30 m, I.D. 0.25, film 0.25 μm). The temperature program started at 40°C for 1 min, ramped up to 162.5°C at 15°C/min, ramped up to 200°C at 40°C /min, and held at 120°C for 1 min.

Elemental sulfur was measured by weighing out approximately 4 g of solid sample (combined samples from ports 2, 3, 5, 6, and 7) and dissolving the sample in 14 mL methanol and mixed by rotating overnight (12–16 h) in an anaerobic chamber (Amend et al., 2004). Sample was filtered (0.45 μM filter) and analyzed on a Dionex HPLC-UV (Thermo Fisher, Sunnyvale, CA, United States) outfitted with a 4.6 × 250 mm, 5 micron Zorbax ODS column (Agilent, Santa Clara, CA, United States) with a methanol mobile phase flowing at 1 mL/min and UV detection at 265 nm. The Sulfur standard was made by dissolving 16 mg of elemental sulfur in 25 mL chloroform and 1 mL of 10% nitric acid and adding methanol to a final volume of 250 mL, followed by 5 min of sonication to dissolve the sulfur. Because the sonication is difficult to perform anaerobically, the column samples were instead extracted for a much longer period of time using the rotation method above.

### Dose Response Analysis

For IC_50_ calculations, actual inhibitor values measured in the influent bottles were averaged over the entire treatment phase and the data were normalized using a sulfide production rate of 0 mmoles per day as 0% and the average of the no treatment column rates over the entire experiment (0.30 mmoles/day) as 100%. Concentrations were then log transformed. Non-linear regression curve fits were created for standard inhibition dose-response curves in GraphPad Prism 6 (GraphPad Software Inc., La Jolla, CA, United States). A Fractional Inhibitory Concentration Index (FICI) based on the IC_50_ values for nitrate and perchlorate treatments was calculated as in Carlson et al. (2015a) and defined as follows: FICI < 0.5 = synergism; FICI = 1–2 indifferent/additive; FICI > 2.0 = antagonism (European Committee for Antimicrobial Susceptibility Testing [EUCAST], 2000).

### Microbial Community Analysis

Solid column samples were thawed and approximately 0.5 g of each sample was added into the bead tube of a Mo Bio Powersoil DNA Isolation Kit. DNA was extracted from the samples following the manufacturer’s protocol. PCR, Illumina library generation, and MOTHUR analysis were performed as in Carlström et al. (2016). Briefly, the 16S rRNA gene was amplified using 2x KAPA HiFi HotStart ReadyMix and 5 μM of the universal MiSeq 16S F (5′ TCG TCG GCA GCG TCA GAT GTG TAT AAG AGA CAG CAG CMG CCG CGG TAA 3′) and MiSeq 16S R (5′ GTC TCG TGG GCT CGG AGA TGT GTA TAA GAG ACA GGA CTA CHV GGG TAT CTA ATC C 3′) primers per 25 μL reaction. PCR conditions were 95°C (3 min); 30 cycles of 95°C (30 s), 64°C (30 s), and 72°C (30 s); and 72°C (10 min). Products were visualized by agarose gel electrophoresis, cleaned up with AMPURE XP beads, indexed with the Illumina Nextera XT index kit and sequenced on an Illumina MiSeq PE 250 platform and de-multiplexed by the UC Davis Genome Center DNA Technologies Core. FASTQ files were analyzed using MOTHUR v. 1.36.1 (Schloss et al., 2009). Forward and reverse reads were merged, sequences were aligned using the SILVA database (Pruesse et al., 2007), and chimeras were removed using UCHIME (Edgar et al., 2011). Sequences were clustered into operational taxonomic units (OTUs) using a 3% dissimilarity cut-off and assigned taxonomic identities using the RDP database.

Statistical analyses of the OTU data were performed using Primer 7 (Clarke and Gorley, 2015). All data were standardized and fourth root transformed, and a Bray Curtis similarity matrix was created. Non-metric multidimensional scaling (nMDS) plots were then generated using the similarity matrix (Clarke, 1993). Similarity clustering on the plots (circles) were created with hierarchical clustering using group average to form a dendogram. SIMPROF was used to test for significant clusters on the dendogram (Clarke et al., 2008). All clusters circled on the nMDS plots were significant by SIMPROF. Means nMDS plots were created by averaging the replicate samples and creating a Bray Curtis similarity matrix from the averaged values. Trajectories were plotted on these plots using the trajectory tool in Primer 7. Similarity percentage (SIMPER) was used to determine the OTUs contributing to the top 10% of the differences between various groupings. The average abundance in the SIMPER output for each OTU was subtracted from the comparison group’s value and positive values (indicating enrichment in that condition) were separated from the negative values (indicating inhibition in that condition). These values were then summed by family or phylum (class for Proteobacteria) and used to create enrichment and inhibition graphs in Excel.

## Results

### Geochemistry

**FIGURE 2:**
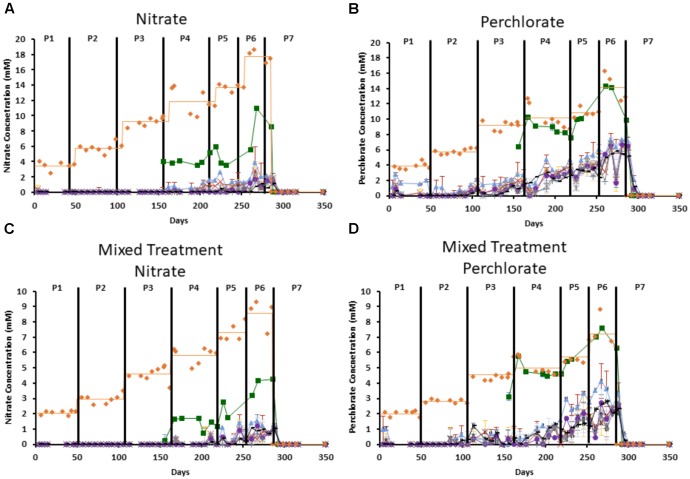
Measured influent concentration of each inhibitor throughout the columns. Orange diamonds represent the influent concentration and the orange line represents the average influent concentration during each treatment phase, green boxes represent port 1, blue triangles represent port 2, red “x”s represent port 3, yellow “^∗^”s represent port 4, purple circles represent port 5, gray “ + ”s represent port 6, and black “-”s represent port 7. Treatment phases (1–7) are indicated by the black lines and labeled as P1–P7. **(A)** Is nitrate concentration in the nitrate only columns, **(B)** is perchlorate concentration in the perchlorate only columns. **(C,D)** Represent the nitrate concentration **(C)** and perchlorate concentration **(D)** in the mixed treatment columns. Error bars represent standard deviation of triplicate samples with the exception of influent, which only has a single measurement at each time point.

Consumption of inhibitor varied by port and treatment, with consistently more nitrate consumed than perchlorate (**Figure 2**). By port 1 (the point where the influent enters the column) in the nitrate only treated columns 32.4–46.5% of the influent nitrate was already consumed and by port 2 (the first solid sampling port) 0–3.3% of the influent concentration remained (**Figure 2A**). In contrast, in the perchlorate only treated columns, very little perchlorate was consumed by port 1 and it was never fully consumed in the column, with 2–38% remaining depending on the treatment phase (**Figure 2B**). In columns receiving the mixed treatment, all the nitrate was consumed by port 2 in the early phases of treatment (phases 1–3), with only 10% of the influent nitrate remaining at the final port even in the highest phase of treatment (phase 6 = 8.58 ± 0.67 mM nitrate treatment, **Figure 2C**). In contrast, the perchlorate component of the mixture was much more recalcitrant and was only completely consumed in phase 1 (2 ± 0.40 mM perchlorate treatment) and by phase 6 almost 34% of the influent perchlorate (7.22 ± 0.93 mM) remained in the effluent (**Figure 2D**). VFAs were completely consumed by port 7, and partially consumed (up to 49.74%) by port 1 in some columns.

**FIGURE 3:**
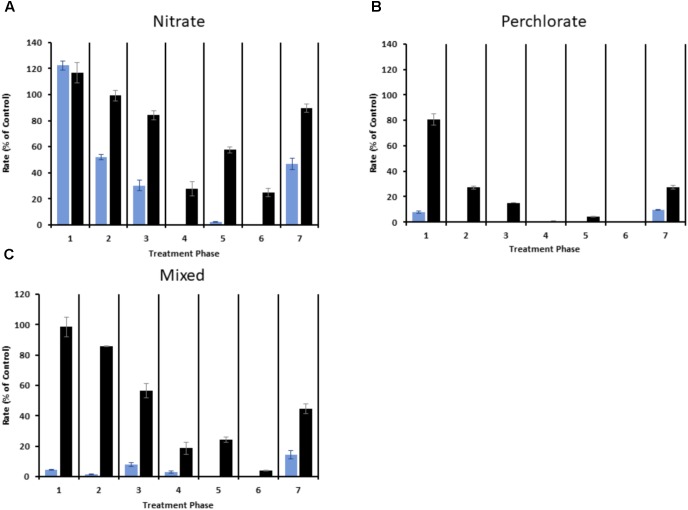
Sulfide production rate expressed as percent of the average rate (in mmoles/day) for the control columns for each treatment phase. Blue bars represent port 2 and black bars represent port 7 (effluent). **(A)** Represents nitrate treated columns, **(B)** represents perchlorate treated columns, and **(C)** represents two of the mixed treatment columns. Error is the standard deviation of triplicate samples for panels A and B and the range of duplicate samples for panel C.

The sulfide production rate decreased as the treatment concentrations increased under all treatment regimes (**Figure 3** and **Supplementary Figure S3**). At nitrate treatment concentrations of 3.67 ± 0.50 mM, nitrate by itself was ineffective at inhibiting sulfide production and measured sulfide production rates ranged from 90.9 ± 6.3% to 122.4 ± 3.5% of control values (**Figure 3A** and **Supplementary Figure S3A**). By phase 2 the sulfide production rates in first sampling point (port 2) had decreased to 51.9 ± 2.2% of to the control columns while effluent (port 7) sulfide remained at 99.2 ± 4.0%. In phase 3 the sulfide production rate in port 2 further dropped to 30.2 ± 4.1% and effluent sulfide also dropped to 84.2 ± 3.4% of the control column. In the 4th treatment phase sulfide production in port 2 had dropped to nearly undetectable levels while all the other ports had dropped to less than 50% of the sulfide production in the control columns. Treatment phase 5 was similar to phase 4 with the only significant change in sulfide production occurring at port 4 (**Supplementary Table S1**). The final phase of treatment (phase 6; 17.75 ± 0.67 mM nitrate treatment) resulted in a significant drop in effluent sulfide (**Supplementary Table S1**). When treatment was removed in phase 7 all sampling ports rebounded with average sulfide production at a single time point ranging from 47.0 ± 4.4% in port 2 to 89.6 ± 3.4% in the effluent (port 7). effluent sulfide was consistently higher than in the other ports, with the exception of treatment phases 4 and 6.

Perchlorate treatment was more effective than nitrate treatment throughout all phases of the study (**Figure 3B**, **Supplementary Figure S3B**, and **Supplementary Table S1**). In treatment phase 1 the sulfide production rate in port 2 and in the effluent fell to 7.6 ± 0.8% and 80.9 ± 4.4% of the control columns respectively. In phase 2, sulfide production rates further fell to undetectable levels in port 2 and to 37.7 ± 1.7% in the effluent. Sulfide production rates further decreased in phases 3, 4, and 5, with treatment phase 6 (14.18 ± 1.61mM perchlorate treatment) completely inhibiting all sulfide production in the columns. When treatment was removed in phase 7 sulfide production rebounded, with rates ranging from 9.8 ± 0.3% to 27.3 ± 1.5% when compared to the control columns and significantly lower than the rebound rates seen in the nitrate treated columns (**Supplementary Table S1**).

In the case of the mixed treatments, two of the triplicate columns showed more sulfide inhibition than the nitrate only columns but less than the perchlorate only columns (**Figure 3**). Similarly to the nitrate treatment, sulfide production was never completely halted in these columns, with the exception of sampling port 2, but sulfide production rates fell to between 1.4 ± 0.4% and 15.3 ± 1.7% of the control column values depending upon the sampling port and treatment phase. In phase 7, sulfide production in these columns rebounded to a rate that was less than the nitrate treatment but higher than perchlorate treatment. One aberrant column of the triplicates (**Supplementary Figure S3D**) treated with mixed treatment responded to treatment better than the perchlorate only treated columns and also barely rebounded in phase 7.

Analysis of the sulfide production rates across the columns throughout the various treatments revealed that the IC_50_ for nitrate treatment was 10.92 mM (95% confidence interval of 9.81–12.0 mM) while the value for perchlorate treatment was 4.85 mM (4.48–5.23 mM). The average for the mixed treatment was 6.43 mM (5.47–7.46 mM). If the anomalous replicate is excluded from the analysis the IC_50_ for the mixed treatment increases to 8.56 mM (range of 2.24–9.93 mM). FICI values for the mixed treatment were 1.9 (1.8–2.0) with all replicates included or 2.5 (2.3–2.7) with the anomalous replicate excluded. These values indicate that the two compounds have additive or potentially antagonistic effects.

Previous studies have suggested that microbial sulfur oxidation might be an important component of nitrate control of souring (Voordouw et al., 1996; Hubert et al., 2009). Furthermore, it has been clearly demonstrated that all dissimilatory perchlorate reducing microorganisms (DPRM) innately oxidize sulfide incompletely to elemental sulfur through a short circuiting of their electron transport respiratory pathway (Gregoire et al., 2014; Mehta-Kolte et al., 2017). To determine the impact of this potential biogeochemical redox cycling on sulfur speciation under the different treatments we monitored the elemental sulfur (S^o^) content in the various columns for each treatment phase after equilibria were established. A small amount of S^o^ was detectable in the crude oil (3.94 ± 0.20 μg/g) used to saturate the column material prior to column packing. In contrast, no S^o^ was detected in the bay sediment used in the column packing material. In the case of the control columns, results indicated that an average concentration of 72.82 ± 15.64 μg/g (average of all treatment phases) was established throughout the operation, far exceeding the content that could be accounted for by column packing materials. These results suggest that the high levels of S^o^ seen during column operation was likely the result of biotic sulfur cycling including incomplete sulfate reduction and sulfide oxidation.

The S^o^ content observed in the nitrate treated columns was not significantly different from that of the control columns at any point during the study (ANOVA, *P* = 0.3093) and overall the S^o^ content in these column sets remained relatively consistent throughout the entire experimental operation. This was expected as most known sulfur-oxidizing nitrate-reducing microorganisms completely oxidize sulfide to sulfate without forming significant amounts of S^o^ (Gevertz et al., 2000; Wang et al., 2005; Cardoso et al., 2006; Gadekar et al., 2006; Tang et al., 2009). In contrast, to both the control and nitrate treated columns, the S^o^ content in the perchlorate and mixed treatment columns increased in the initial phases of treatment and peaked in phase 2 (211.66 and 234.26 μg/g for the perchlorate and mixed treatments respectively) after which the S^o^ content quickly dropped and by phase 4 S^o^ concentrations were back at levels equivalent to those observed in the control and nitrate treated columns (**Supplementary Figure S4** and **Supplementary Table S2**). The observed dynamics of the S^o^ content in columns amended with perchlorate is consistent with sulfide oxidation to S^o^ by DPRM combined with inhibition of SRM activity.

### Microbial Community

**FIGURE 4:**
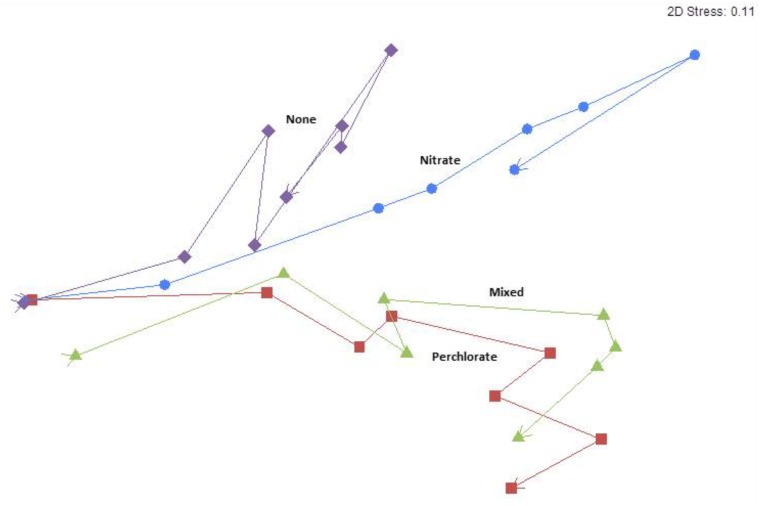
Means non-metric multidimensional scaling plot of each treatment over time/treatment phase from pre-treatment to phase 7. Arrows indicate directionality from Pretreatment toward phase 7 and each point represent an average value for all samples from that treatment phase. Blue circles represent the nitrate treatment, red squares represents perchlorate treatment, green triangles represents mixed treatments, and purple diamonds represent no treatment. The stress value in the upper right hand corner indicates goodness of fit to the data with 0 representing a perfect fit and 0.3 representing a random fit.

The nMDS analysis revealed grouping by treatment with samples diverging as treatment concentrations increased over the treatment phases (**Figure 4**). The trajectory over treatment phase of the perchlorate treated samples and the mixed treatment samples closely resembled each other, even with the aberrant column included. The nitrate treated samples showed a unique trajectory, which was similar to the no treatment control in early phases and diverged in later phases of higher treatment concentrations. Removing treatment in the final phase did cause a community shift but the community did not revert to a similar structure to that of the initial or control communities. There is also a clear trend of greater separation of the community make-up of the treatments as treatment concentration increases throughout the treatment phases and when the columns are allowed to re-sour in phase 7 the treatments remain separate from each other and do not return to the pretreatment community (**Supplementary Figure S5**). The microbial community from the anomalous mixed treatment column is different than the other two replicates but since the differences are all in unclassified OTUs, there is no clear indication of how the different community composition might relate to the enhanced inhibition of sulfide production observed in this column (**Supplementary Figure S6**).

**FIGURE 5:**
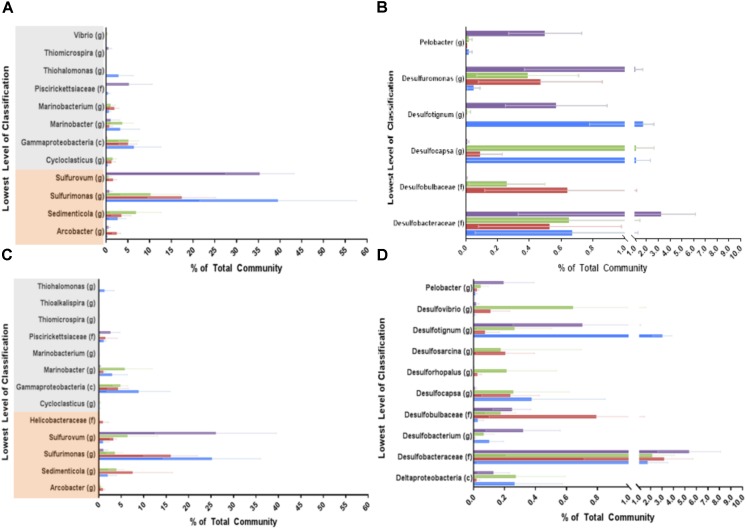
Simper selected OTUs representing the top 10% of the difference between each treatment and the untreated columns during phases 6 (the highest treatment concentration, **A,B**) and 7 (re-souring, **C,D**). OTUs were summed by the lowest level of classification available; genus (g), family (f), or class (c) and represented here. Blue bars represent the nitrate treatment, red bars represent the perchlorate treatment, green bars represent the mixed treatment, and purple bars represent the untreated columns. Error bars represent the propagated error for each phylum/class based on the OTU standard deviations across all samples (ports) during the treatment phase. **(A,C)** Represent the Gammaproteobacteria (highlighted in gray) and Epsilonproteobacteria (highlighted in orange). **(B,D)** Represent the Deltaproteobacteria.

To identify organisms responsible for the community differences due to treatment, similarity percentage (SIMPER) was used to identify the OTUs contributing to the top 10% of the differences between the treatment groups during high treatment (phase 6: **Figures 5A,B**, **Supplementary Figure S7A**, and **Supplementary Table S3**). These OTUs were then summed by phylum (or class for the Proteobacteria). In both phases (6 and 7) the largest contributing phyla for all treatments were Gammaproteobacteria, Epsilonproteobacteria, Deltaproteobacteria, Unclassified Bacteria, and Bacteroidetes (**Supplementary Table S1**). In phase 6 Gammaproteobacteria and Epsilonproteobacteria were some of the most dominant groups contributing to the differences between treatment (**Figure 5A** and **Supplementary Figure S7A**). Within the Epsilonproteobacteria class, the *Sulfurimonas* and *Sedimenticola* genera were highly enriched by all treatments compared to control columns. These sulfur-oxidizing organisms are known to also be able to use nitrate or perchlorate as electron acceptors (Takai et al., 2006; Carlstrom et al., 2015). *Sulfurovum*, also containing sulfur-oxidizing representatives, was the most dominant genus in the untreated samples. This genus was inhibited by all treatments, and was nearly eradicated in the nitrate only columns. *Arcobacter* was enriched only under perchlorate treatment, which was not surprising since some *Arcobacter* species can use perchlorate as an electron acceptor (Carlström et al., 2013).

In the Gammaproteobacteria class, *Cycloclasticus* was enriched by all treatments (**Figure 5A**). Members of this hydrocarbon degrading group can reduce nitrate and are closely related to sulfur-oxidizing organisms (Dyksterhouse et al., 1995; Chung and King, 2001). Unclassified Gammaproteobacteria and members of the *Marinobacterium* genus were also highly enriched under all treatments. These organisms were presumably nitrate or perchlorate reducing organisms, as members of the *Marinobacterium* genus are known to reduce nitrate, although no perchlorate reducing member has yet been identified (Chang et al., 2007; Huo et al., 2009). The known hydrocarbon degrading and nitrate-reducing group, *Marinobacter*, was enriched specifically in treatments containing nitrate (Gauthier et al., 1992; Yoon et al., 2003). *Piscirickettsiaceae* and *Thiomicrospira* were both inhibited by all treatments. *Thiohalomonas* was highly enriched by nitrate treatment but appeared to be inhibited by perchlorate as it was not detectable in perchlorate or mixed treatments. This group includes organisms that can use thiosulfate as an electron donor and nitrate as an acceptor (Sorokin et al., 2007). *Vibrio* was also enriched by nitrate treatment, which is also unsurprising as *Vibrio* species have long been known to have the ability to respire nitrate (Macfarlane and Herbert, 1982; Proctor and Gunsalus, 2000).

Also of specific interest is the Deltaproteobacteria class, which includes the vast majority of known sulfate reducing bacteria (**Figure 5B**). *Desulfobulbaceae*, which can grow by sulfur oxidation (Malkin et al., 2017) or sulfate reduction (Kuever, 2014) were enriched in both treatments involving perchlorate. Enrichment of this genus has been previously observed in marine sediment columns containing perchlorate (Engelbrektson et al., 2014). Interestingly, they were not enriched by nitrate even though nitrate reducing *Desulfobulbaceae* have been identified (Malkin et al., 2017). *Desulfocapsa*, a member of *Desulfobulbaceae* and capable of elemental sulfur disproportionation (Janssen et al., 1996; Finster et al., 1998), was also enriched by all treatments, though it appears to be more enriched by nitrate than perchlorate. *Desulfotignum*, a known toluene degrader and sulfate reducer (Ommedal and Torsvik, 2007), was inhibited by perchlorate but not nitrate. Interestingly, the sulfate reducing *Desulfobacteraceae*, were not significantly inhibited by any specific treatment. However, the sulfur reducing *Desulfuromonas* and the sulfur cycling *Pelobacter* (Lovley et al., 1995; Haveman et al., 2008) were both inhibited by all treatments.

The same analysis was performed on phase 7 samples to see what changes occurred after treatment was removed and the columns were allowed to re-sour (**Figures 5C,D**, **Supplementary Figure S7B**, and **Supplementary Table S1**). Many of the same dominant Phyla/Classes noted in phase 6 were dominant in phase 7 as well. However, Firmicutes appeared to play a bigger role after re-souring and Bacteroidetes appeared to play a lesser role as they returned to nearly identical levels observed in the untreated columns (**Supplementary Figure S7**). Gammaproteobacteria and Epsilonproteobacteria, however, were still the most dominant groups. As expected, Deltaproteobacteria increased in all the treated columns when the treatments were removed.

Within the Epsilonproteobacteria class, *Sulfurovum* increased in the formerly treated columns compared to treatment phase 6 but did not recover to the levels seen in the untreated columns (**Figures 5A,C**). *Sulfurimonas* decreased in the nitrate treatment but did not change much in the other treatments. *Sedimenticola* and *Arcobacter* didn’t appear to appreciably change in abundance. The Gammaproteobacteria showed no apparent significant change from high treatment to re-soured.

There were, however, changes in the Deltaproteobacteria (**Figures 5B,D**). The sulfate reducing genera, *Desulfovibrio*, *Desulfosarcina*, *Desulforhopalus*, and *Desulfobacterium*, played a role in re-souring in the treated columns (phase 7) but not in the high treatment phase (phase 6). All of these groups were enriched, with the exception of *Desulfobacterium*, which was inhibited compared to the control. *Desulfobacteraceae* appeared to be the only group that recovered back to untreated control levels in all the treated columns. *Desulfocapsa* is the only genus that continued to be enriched in treated columns even after treatment was removed. Other groups such as *Desulfotignum* and *Desulfobulbaceae* were enriched in some treatments but not others, just as in treatment phase 6. *Pelobacter* remained inhibited in treated columns even after treatment was removed.

## Discussion

The results of these studies demonstrate that combining perchlorate and nitrate in equimolar amounts is an effective strategy for inhibition of sulfidogenesis that is inherently more effective than nitrate treatment alone. Interestingly, geochemical and microbial community structure analysis revealed that the combined inhibitors acted additively, but performed more similarly to the independent perchlorate treatment rather than the nitrate treatment. This suggests that the perchlorate component of the mixture was the dominating control mechanism which may leave room for formulation optimization for improved potency in comparison to nitrate alone in addition to cost effectiveness. Previous work revealed the efficacy of competitive inhibitors, specifically nitrate and perchlorate, at combatting oil reservoir souring (Myhr et al., 2002; Hubert et al., 2003; Hubert and Voordouw, 2007; Gieg et al., 2011; Engelbrektson et al., 2014, 2018; Carlson et al., 2015a). However, only a few studies have examined the benefit of mixing multiple inhibitors. Greene et al. (2006) looked at the synergy between nitrite and various antimicrobial agents or between the antimicrobial agents themselves and discovered that a number of the compounds were, synergistic, but others were indifferent or antagonistic (Greene et al., 2006). Carlson and coworkers were the first to look at synergy between different competitive inhibitors in a quantifiable systematic high throughput manner using a pure culture system and found that perchlorate and nitrate were additive, while nitrite was synergistic with perchlorate and chlorite was synergistic with nitrate (Carlson et al., 2015a,b, 2017). However, the dynamics of a community based column system are very different than those at play in a pure culture or batch culture study. In particular, the competition for an electron acceptor can lead to biocompetitive exclusion mechanisms of inhibition (Youssef et al., 2009; Gieg et al., 2011; Carlson et al., 2015a; Youngblut et al., 2016). Furthermore, production of reactive intermediates (e.g., nitrite, chlorite, molecular oxygen) by nitrate reducing microorganisms and perchlorate reducing microorganisms likely contribute to the mechanism of these inhibitors (Callbeck et al., 2013; Engelbrektson et al., 2014). Additionally components of the packing material (e.g., iron) may chemically react with these reactive intermediates and with sulfide creating indirect microbially driven geochemical redox cycles (Engelbrektson et al., 2014). Since nitrate is often preferentially used before perchlorate in microbial communities (Coates and Achenbach, 2004; Coates and Jackson, 2009; Wang et al., 2018), a central hypothesis in this study was that mixing the two treatments in equimolar concentrations would result in preferential use of the nitrate as an electron acceptor, allowing for further penetration of the perchlorate into the column matrix and potentially increasing the effectiveness of the treatment. A feature of this hypothesis is that stratification of nitrate and perchlorate reducing communities should lead to different community structures in the columns and differences in the column geochemical profile.

Our results indicated that nitrate was indeed preferentially utilized by the community (**Figure 2**) and, thus, perchlorate was detected further in the column matrices, especially under the higher treatment concentrations. However, while better than nitrate, the mixed treatment was not consistently more effective at inhibiting sulfide production than perchlorate treatment alone at the same total concentration. This was evidenced by the fact that although sulfide production in one of the mixed treatment columns was lower compared to those treated with perchlorate alone, the other two replicates of the mixed treatment columns were less inhibited throughout the experiment (**Figure 3**). The mixed treatment did, however, unfailingly result in lower rates of sulfide production at the same total treatment concentration than nitrate. The dose-response analysis of the geochemical data show very similar results to those seen in previous pure culture studies where a FICI of 1.8 was calculated, indicating an additive effect (Carlson et al., 2015a). We obtained a nearly identical FICI of 1.9 when all three replicates were included, although a higher FICI of 2.5, indicating slight antagonism between perchlorate and nitrate inhibitory potency, was obtained when the anomalous replicate was excluded.

Another factor at play was the increasing concentration of inhibitor from ∼5 mM to ∼20 mM as the study progressed which allowed us to calculate IC_50s_ and FICIs from the data to enable direct comparison of the different inhibitors. The study design also enabled us to identify the concentrations at which a decrease in the sulfide production rate was seen at each port along the column. The most affected port was port 2 (the earliest solid sampling point), while all the subsequent ports showed very similar effects to each other (**Figure 3**). This suggested that primary impact of the individual inhibitors was occurring in the first 3 inches of the column before port 3. In general, this is not unexpected as this location close to the injection point is expected to have the highest inhibitor concentration. In the combined treatment, there was a greater difference in sulfide production at the more distant ports compared to the nitrate only treatment, supporting the theory that the mixed inhibitors had a greater zone of impact that the nitrate treatment alone, although this trend was only obvious in two out of the three columns as one column behaved anomalously (**Figure 3**). Also, it is very clear that in contrast to the perchlorate only treatment, the nitrate and mixed treatments never completely eliminated sulfate reduction in the columns, even at a concentration of nearly 20 mM. Average sulfide production in the perchlorate columns was very low (0 – 4.25%) relative to the controls across the length of the columns by phase 5, and by phase 6 sulfide production was completely eradicated.

Since microbial community analyses on previous column studies indicated that nitrate treated columns support a very different community structure than perchlorate treated columns (Engelbrektson et al., 2014, 2018) it was presumed that the community of the mixed treatment columns would resemble a mixture of those seen in each of the single treatment columns. Surprisingly, however, the trajectory of the mixed treatment community closely followed that of the perchlorate treatment alone despite receiving only half the concentration of perchlorate at any measured point, indicating that the effect of perchlorate on community structure was a stronger selective force compared to that of nitrate (**Figure 4**).

The increasing inhibitor concentrations also affected the microbial communities. The community trajectories show that, although the untreated columns do change over time, these changes are not as directional or as extreme as the changes seen in the treated columns as the inhibitor concentrations increased (**Figures 4**, **5**). It took only until phase 3 (9.2 ± 0.5 mM treatment) for the community in the nitrate treated columns to diverge, but the perchlorate and mixed treatment columns didn’t diverge until phase 5 (10.8 ± 0.5 mM total concentration) and never diverged from each other. This could potentially be due to the rarity of perchlorate reducing organisms in the environment versus nitrate reducing organisms, which are much more likely to be present in higher abundances initially in bay sediment.

The final factor tested was the extent of re-souring after removal of treatment (at this point all of the treated columns had received nearly a year of continuous treatment). All the columns re-soured to some extent, but in the nitrate treated columns the sulfide rebounded to a rate 89.6% of the control while in the perchlorate treated columns it only rebounded to 27.3% (**Figure 3**). As expected, the level of re-souring in the combined treatment fell between those of the two single treatments (44.8%). Oil reservoirs often undergo periods during which no treatment is applied (shut-in) to service the surface facilities, so treatments that minimize re-souring during subsequent shut-in periods would be of great value.

The microbial community of the mixed treatment closely resembles that of the perchlorate treated columns. This is important because it lends credence to the concept that perchlorate is the primary active ingredient causing inhibition in the mixed treatment columns. This is also supported by the fact that nitrate does not make it very far into the columns before it is completely removed by nitrate reducing organisms. The community makeup indicates that perchlorate has some important differential effects on the community that are not seen under nitrate treatment alone. Perchlorate reducing organisms are enriched when perchlorate is present and all known dissimilatory perchlorate reducing bacteria have the innate ability to oxidize sulfide to elemental sulfur (Gregoire et al., 2014; Mehta-Kolte et al., 2017). Elemental sulfur concentrations were higher in columns containing perchlorate, confirming the results of previous studies that indicated that perchlorate reduction is coupled to sulfide bio-oxidation (Gregoire et al., 2014; Mehta-Kolte et al., 2017). Since sulfide is the electron donor in this reaction, the ultimate sulfur concentration will be directly dependent on the residual sulfide concentration after SRM activity is inhibited. As no further sulfide production is occurring, elemental sulfur concentrations in a dynamic system are expected to peak shortly after perchlorate treatment is initiated after which the S^o^ concentrations should decrease back to background levels. This is consistent with the observed results which showed an initial increase in elemental sulfur concentration in the perchlorate only and combined treatment columns from phase 1 to phase 2, where sulfide is still present, and a subsequent decrease in elemental sulfur concentration back to control column levels after phase 4, when sulfide was completely removed.

Members of the *Desulfotignum* genus, one of the most abundant sulfate reducing genera in this system, are only highly inhibited in columns treated with perchlorate. Nitrate, on the other hand, enriched for sulfur disproportionating organisms, which can actually lead to the production of sulfide through this novel form of sulfur cycling. When treatment was removed from the columns there were few major changes in the community composition, suggesting that the activity seen during the re-souring phase was due mainly to the presence of sulfate reducing organisms that persisted in the columns during treatment. Even after the cessation of treatment, the mixed treatment community remained very similar to that of the perchlorate treated columns, indicating that perchlorate had a lasting effect on community structure. The results presented here show that perchlorate alone or a perchlorate/nitrate mix are both better choices for combating souring than nitrate alone. These treatments not only inhibit souring to a greater degree than nitrate but also show a greater potential at inhibiting re-souring during shut-in periods. Additionally important changes occur in the microbial community when perchlorate is added that make the community less favorable to sulfate reduction. These same trends hold true when the columns were allowed to re-sour, and the columns treated with perchlorate (alone or in combination with nitrate) re-soured to a lesser extent than equimolar nitrate treatment alone. Combining these two treatments may provide an economic benefit compared to using either one independently at higher concentrations. Additionally, enriching for both perchlorate reducing and nitrate reducing microorganisms may provide a more diverse barrier against both rebound and resistance of sulfate reducing bacteria resulting in prolonged treatment efficacy.

## Author Contributions

JC designed and guided the project, and co-authored the text. AE designed the experimental plan, performed the daily operations of the experiment, and co-authored the manuscript. VB, MY, and GS aided in daily sample collection and analysis. YL advised on analytical chemistry operations and performed some analysis. IF aided in community computation analysis. HC aided in IC_50_ calculations and experimental design.

## Conflict of Interest Statement

JC holds a patent on the application of perchlorate to the treatment of souring. The remaining authors declare that the research was conducted in the absence of any commercial or financial relationships that could be construed as a potential conflict of interest.
