# Supplementary material for: Mitigating Sulfidogenesis With Simultaneous Perchlorate and Nitrate Treatments
Source: Front Microbiol. 2018 Oct 4;9:2305. doi: 10.3389/fmicb.2018.02305 (PMC6180152; doi:10.3389/fmicb.2018.02305)
Supplement: Supplementary file 1 [file Table_1.docx]

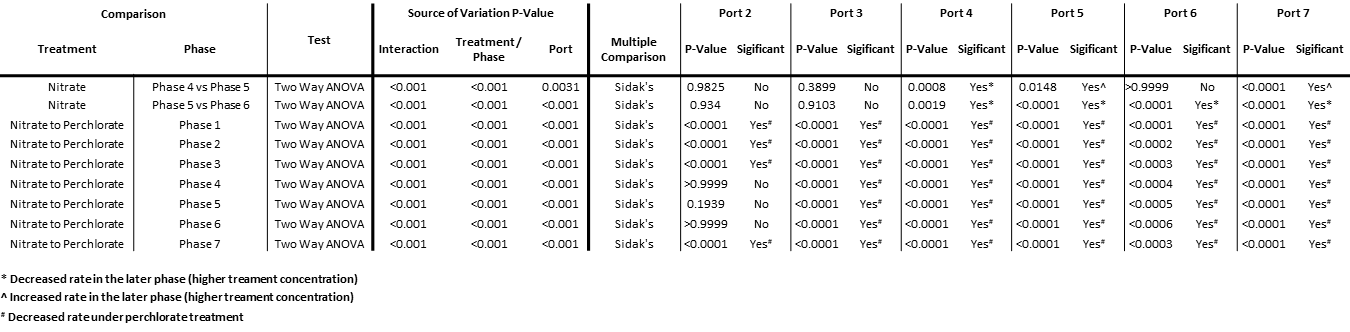


**Table S1:** Summary of the statistical tests and results for the sulfide production rate data for all treatments.
