# Supplementary figures and images for: Mitigating Sulfidogenesis With Simultaneous Perchlorate and Nitrate Treatments
Source: Front Microbiol. 2018 Oct 4;9:2305. doi: 10.3389/fmicb.2018.02305 (PMC6180152; doi:10.3389/fmicb.2018.02305)

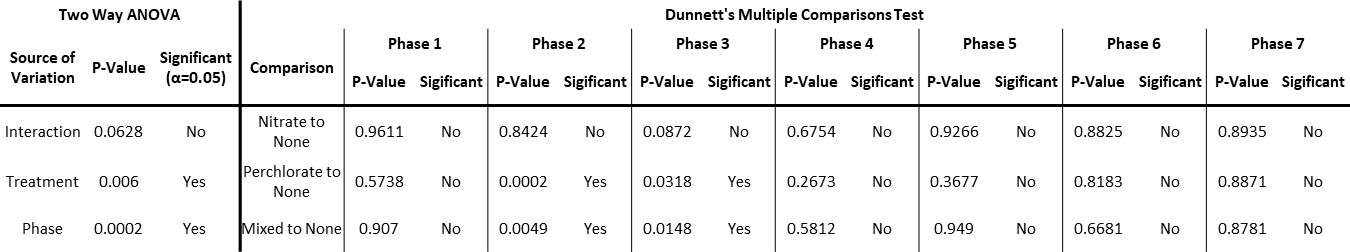


**Table S2:** Summary of the statistical tests and results for the sulfur data for all treatments.

Supplement: Supplementary file 2 [file Table_2.docx]

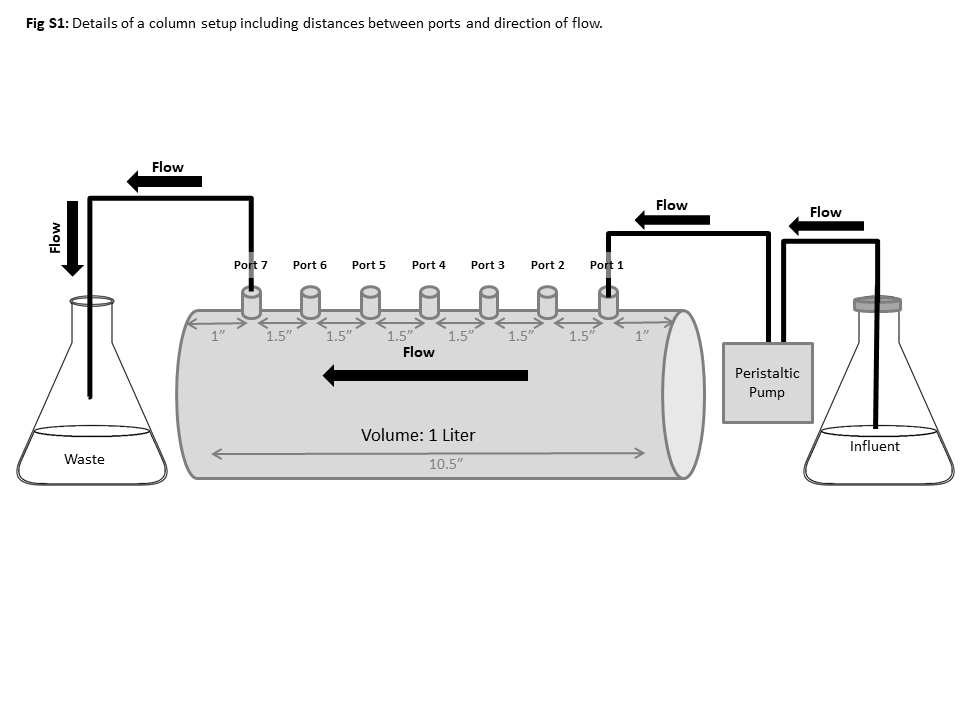

Supplement: Supplementary file 4 [file Image_1.tif]

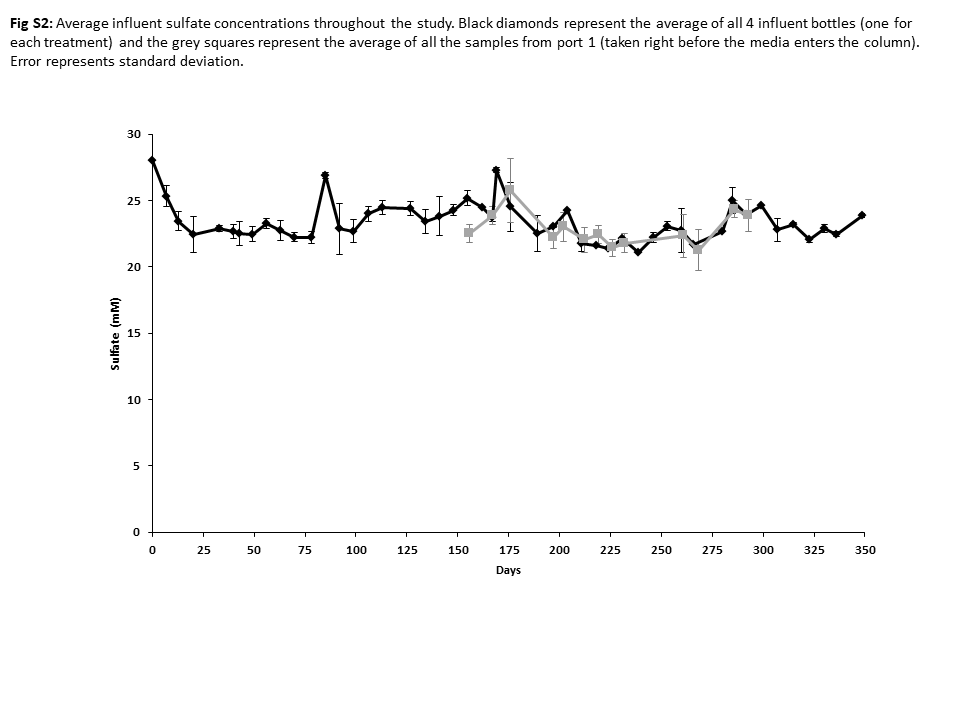

Supplement: Supplementary file 5 [file Image_2.tif]

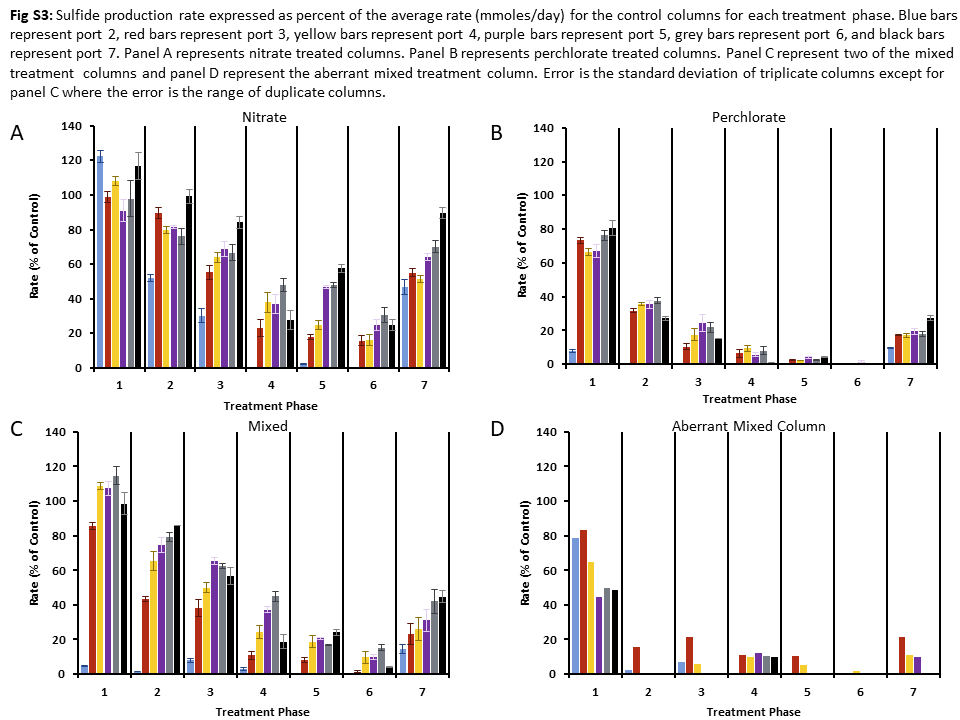

Supplement: Supplementary file 6 [file Image_3.tif]

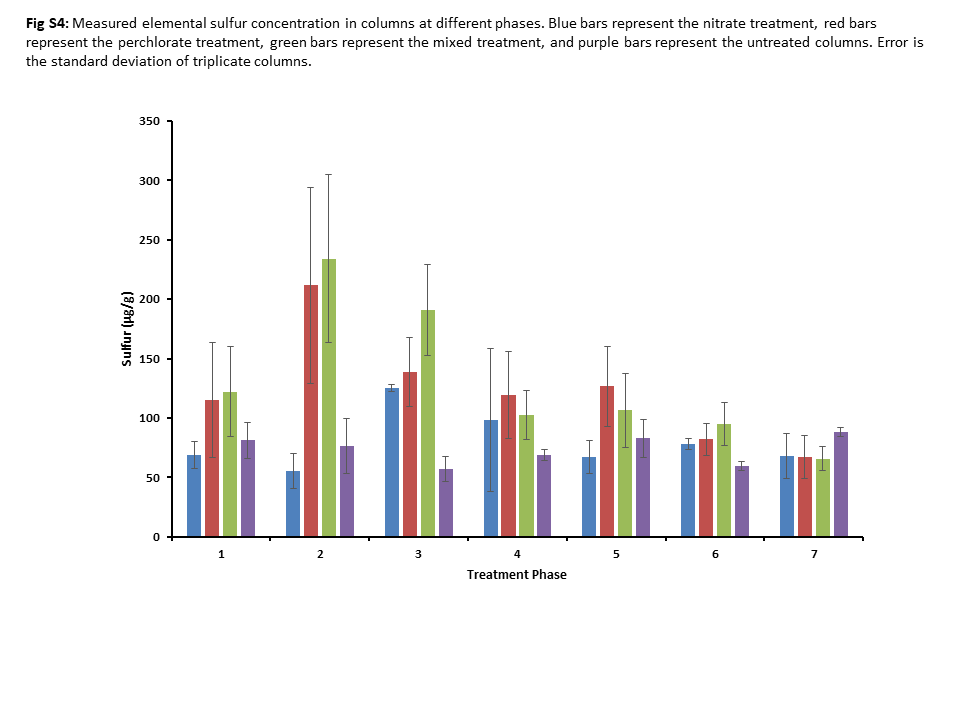

Supplement: Supplementary file 7 [file Image_4.tif]

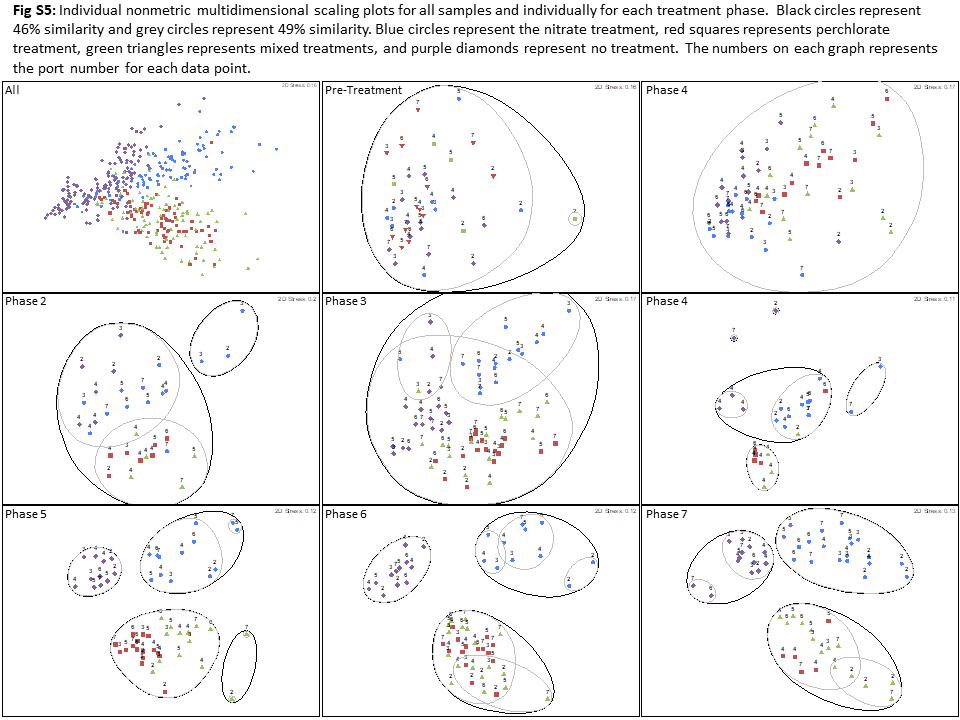

Supplement: Supplementary file 8 [file Image_5.tif]

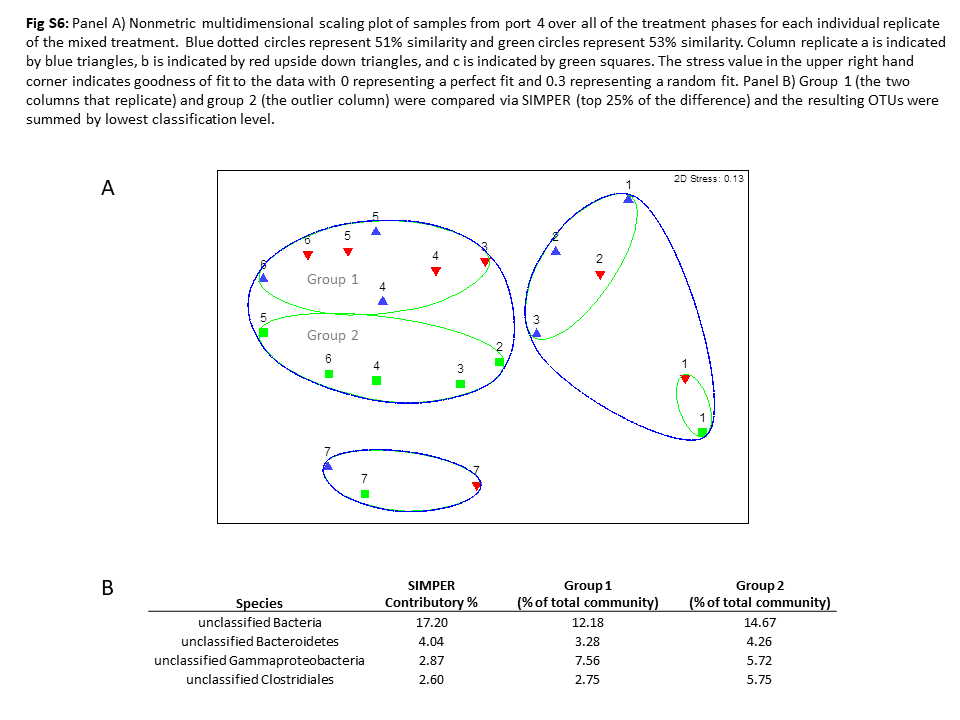

Supplement: Supplementary file 9 [file Image_6.tif]

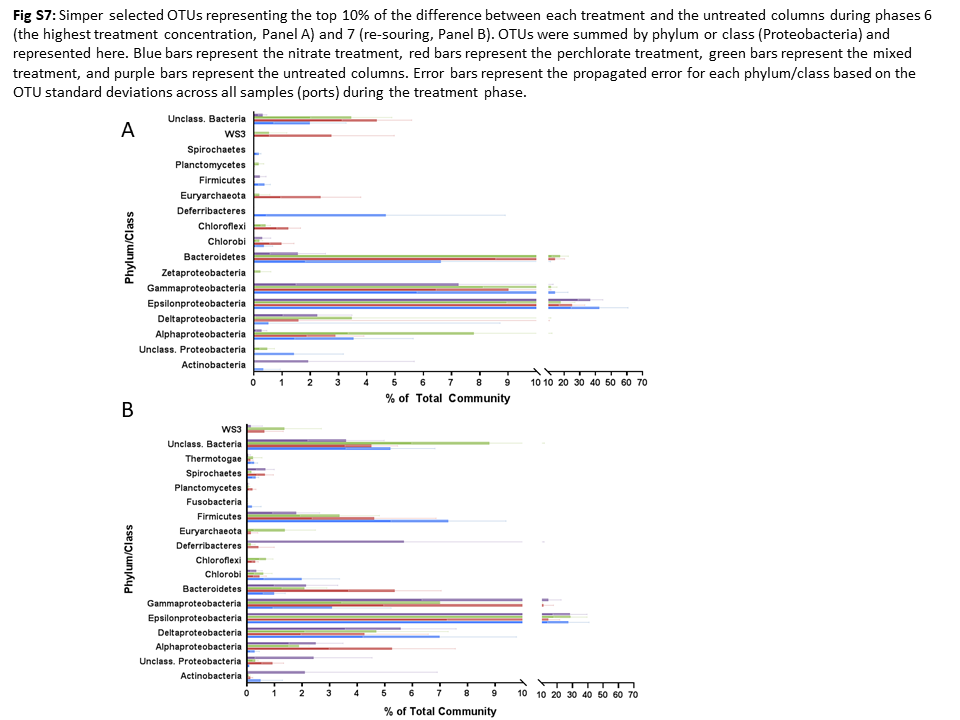

Supplement: Supplementary file 10 [file Image_7.tif]
