# Supplementary material for: Mitigating Sulfidogenesis With Simultaneous Perchlorate and Nitrate Treatments
Source: Front Microbiol. 2018 Oct 4;9:2305. doi: 10.3389/fmicb.2018.02305 (PMC6180152; doi:10.3389/fmicb.2018.02305)
Supplement: Supplementary file 3 [file Table_3.docx]

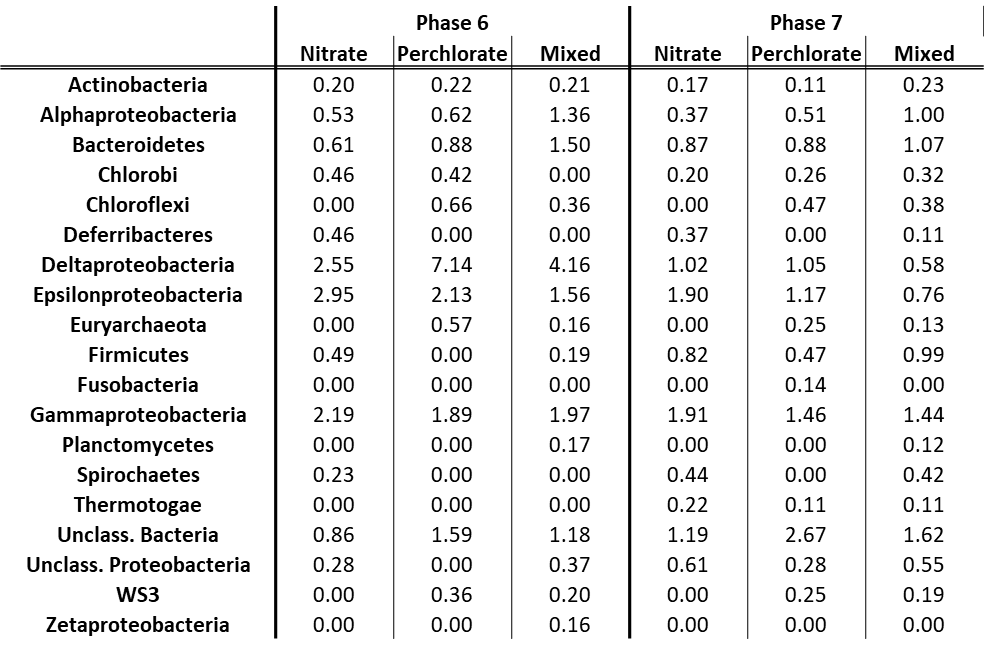


**Table S3:** Contributory percentages for each phylum or class (for Proteobacteria) for each treatment during treatment phases 6 and 7.
